# Neuroanatomical mapping of the lumbosacral spinal cord in individuals with chronic spinal cord injury

**DOI:** 10.1093/braincomms/fcac330

**Published:** 2022-12-19

**Authors:** Samineh Mesbah, April Herrity, Beatrice Ugiliweneza, Claudia Angeli, Yury Gerasimenko, Maxwell Boakye, Susan Harkema

**Affiliations:** Kentucky Spinal Cord Injury Research Center, University of Louisville, Louisville, KY 40202, USA; Kentucky Spinal Cord Injury Research Center, University of Louisville, Louisville, KY 40202, USA; Department of Neurological Surgery, University of Louisville, Louisville, KY 40202, USA; Kentucky Spinal Cord Injury Research Center, University of Louisville, Louisville, KY 40202, USA; Department of Neurological Surgery, University of Louisville, Louisville, KY 40202, USA; Department of Health Management and Systems Science, University of Louisville, Louisville, KY 40202, USA; Kentucky Spinal Cord Injury Research Center, University of Louisville, Louisville, KY 40202, USA; Department of Bioengineering, University of Louisville, Louisville, KY 40292, USA; Frazier Rehabilitation Institute, University of Louisville Health, Louisville, KY 40202, USA; Department of Physiology and Biophysics, University of Louisville, Louisville, KY 40202, USA; Pavlov Institute of Physiology, Russian Academy of Sciences, St. Petersburg 199034, Russia; Kentucky Spinal Cord Injury Research Center, University of Louisville, Louisville, KY 40202, USA; Department of Neurological Surgery, University of Louisville, Louisville, KY 40202, USA; Kentucky Spinal Cord Injury Research Center, University of Louisville, Louisville, KY 40202, USA; Department of Neurological Surgery, University of Louisville, Louisville, KY 40202, USA; Frazier Rehabilitation Institute, University of Louisville Health, Louisville, KY 40202, USA

**Keywords:** spinal cord injury, neuromodulation, MRI, epidural stimulation

## Abstract

With emerging applications of spinal cord electrical stimulation in restoring autonomic and motor function after spinal cord injury, understanding the neuroanatomical substrates of the human spinal cord after spinal cord injury using neuroimaging techniques can play a critical role in optimizing the outcomes of these stimulation-based interventions. In this study, we have introduced a neuroimaging acquisition and analysis protocol of the spinal cord in order to identify: (i) spinal cord levels at the lumbosacral enlargement using nerve root tracing; (ii) variability in the neuroanatomical characteristics of the spinal cord among individuals; (iii) location of the epidural stimulation paddle electrode and contacts with respect to the spinal cord levels at lumbosacral enlargement; and (iv) the links between the anatomical levels of stimulation and the corresponding neurophysiological motor responses. Twelve individuals with chronic, motor complete spinal cord injury implanted with a spinal cord epidural stimulator were included in the study (age: 34 ± 10.9 years, sex: 10 males, 2 females, time since injury: 8.2 ± 9.9 years, American Spinal Injury Association Impairment Scale: 6 A, 6 B). High-resolution MRI scans of the spinal cord were recorded pre-implant. An analysis of neuroanatomical substrates indicates that the length of the spinal column and spinal cord, location of the conus tip and the relationship between the spinal cord levels and vertebral levels, particularly at the lumbosacral enlargement, are variable across individuals. There is no statistically significant correlation between the length of the spinal column and the length of the spinal cord. The percentage of volumetric coverage of the lumbosacral spinal cord by the epidural stimulation paddle electrode ranges from 33.4 to 90.4% across participants. The location of the spinal cord levels with respect to the electrode contacts varies across individuals and impacts the recruitment patterns of neurophysiological responses. Finally, MRI-based spinal cord modelling can be used as a guide for the prediction and preplanning of optimum epidural stimulation paddle placement prior to the implant surgery to ensure maximizing functional outcomes. These findings highlight the crucial role that the neuroanatomical characteristics of the spinal cord specific to each individual play in achieving maximum functional benefits with spinal cord electrical stimulation.

## Teaser

Mesbah *et al*. proposed an image-based approach for neuroanatomical mapping of the lumbosacral spinal cord in individuals with spinal cord injury and showed a considerable amount of variability of neuroanatomical substrates of the spinal cord in humans highlighting the importance of personalized spinal cord modelling for neuromodulation applications in this population.

## Introduction

Spinal cord injury (SCI) caused by acute physical trauma often results in spinal cord lesions at the site of injury, while the rest of the spinal cord remains fairly intact.^1^ Damage to the spinal cord is complex and can affect the body with varying degrees of sensorimotor and autonomic dysfunction.^2^ While spontaneous recovery after injury is variable across individuals, the activation of vital spinal networks below the level of injury in a task-dependent manner can promote plasticity-induced functional and physiological gains.^3^ The use of spinal cord electrical stimulation, with electrodes placed epidurally, induces an electric field sufficient to increase the excitability of relevant neuronal circuits in the intact portion of the lumbosacral enlargement (LSE) and is capable of not only driving voluntary motor activities^4–9^ but also restoring multi-system autonomic improvements in individuals with chronic SCI.^10–17^ Since the aetiology, pathology and secondary consequences of SCI are very diverse, studies in both clinical and pre-clinical models of SCI have focused on evaluating the characteristics of the lesion at the site of injury as a predictor of recovery.^18,19^ However, there is a lack of information regarding the relationship between various neuroanatomical factors in the intact portion of the spinal cord, particularly at LSE, which is the target of epidural stimulation, and the degree of functional recovery in the context of spinal cord epidural stimulation (scES).

We have recently reported that 20 individuals with chronic SCI, classified by the American Spinal Injury Association (ASIA) Impairment Scale (AIS) as AIS A or B, were able to voluntarily perform motor tasks in the presence of scES.^20^ We examined possible links between the demographic and the radiographic factors that may contribute to the extent of volitional recovery after scES implant and training. We reported that there were no direct correlations between the extent of volitional outcomes with age, sex, time since injury, AIS score or neurological level of the injury as well as MRI-based factors related to the injury, such as the length of severe myelomalacia and the amount of spinal cord atrophy above the site of injury. The only factor that significantly correlated with the extent of lower extremity voluntary movement was the amount of coverage that scES paddle electrode encompassed at the LSE. These findings highlight the crucial role that the neuroanatomical characteristics of the spinal cord specific to each individual play in achieving maximum functional benefits with scES. Furthermore, our results suggest that regardless of the severity of injury, based from a radiographic standpoint, individuals may gain functional benefits from scES if the placement of the implanted paddle array is optimized. Neuroanatomical inter-subject variabilities such as volumetric size and length of the spinal cord; length, location, and the angle of the nerve roots, i.e. dorsal roots and rootlets entry zone, with respect to the stimulation site; and the relationship between the spinal cord levels and the vertebral levels at the LSE and conus tip are often not considered or reported in the applications of spinal cord stimulation (epidural or transcutaneous) targeting functional recovery in individuals with SCI. The goal of this study is to use computational modelling and quantitative image analysis to obtain a deeper understanding of the neuroanatomical substrates of the spinal cord and functional properties of the excitable tissues in humans, as well as improving the surgical implantation protocol for scES. We hypothesized that there would be neuroanatomical variabilities across individuals, but targeting similar spinal cord segments with scES would result in similarities in the recruitment patterns of motoneuron pools across participants.

## Materials and methods

### Participants

Twelve individuals with clinically diagnosed chronic, motor complete SCI were enrolled in activity-based training studies using scES, performed at University of Louisville. All research participants were over 21 years of age at the time of scES implant and met the following inclusion criteria: non-progressive SCI at the cervical and upper thoracic spinal cord, AIS A or B, and at least 2 years post injury with no medical conditions unrelated to SCI at the time of implant. All participants provided written informed consent about the purpose of the study and publication of the findings, which was approved by the Institutional Review Board at University of Louisville. Six participants were implanted with the Medtronic RestoreAdvanced™ stimulation (voltage-based stimulation) system and six participants were implanted with the Medtronic Intellis™ stimulation (current-based stimulation) system. Both Medtronic systems use 16-electrode Specify® 5-6-5 leads.

### High-resolution 3D spinal cord MRI

MRI 2D scans of all levels of the spine with high spatial resolution were recorded prior to scES implant surgery using Siemens 3.0 T Magnetom Skyra in sagittal and axial planes. Sagittal images were obtained in two or three separate sequences (depending on the height of the participant) to cover the whole spine from the foramen magnum to the end of sacral region. These images were obtained with a large field of view and were reviewed by the radiologist and neurosurgeon to screen for syrinxes, significant stenosis, scoliosis, level of injury and stabilizing treatment and related surgical changes over time. Typical parameters for sagittal images are as follows:

TR/TE/FA/Thick/ETL/Re_Matrix/PFOV/NSA/BW/Pixal/AQ_matrix/%samp/PE=3000/74/160/∼3×3.45/17/320×320/100%/2/600/1.125×1.125/320×240/75/442

where TR is the repetition time, TE is the echo time, Thick is the slice thickness, ETL is the echo train length, Re_Matrix is the reconstruction matrix, PFOV is the % phase field of view, NSA is the number of signal averages, BW is the bandwidth, Pixal is the pixel dimensions, AQ_matrix is the acquisition matrix, % samp is the % sampling (or partial Fourier) and PE is the number of Phase 3 encodes.

Axial images were obtained using T2 Turbo Spin Echo in four to five separate sequences (depending on the height of the participant) with a focused field of view typically from cervical, upper thoracic, mid thoracic, lower thoracic-upper lumbar and lower lumbar-sacral levels. Axial images were obtained with 3 mm thickness and 0 mm gap. The axial images were used to measure the cross-sectional area of the spinal cord at different vertebral levels and to reconstruct a 3D individual-specific model of the LSE necessary for anatomical mapping of the L1–S1 spinal cord segments (described below). Example parameters are:

TR/TE/FA/Thick/ETL/Re_Matrix/PFOV/NSA/BW/Pixal/AQ_matrix/%samp/PE=3280/82/121/3×3/28/512×512/100%/2/610/0.35×0.35/256×179/70/252

It should be noted that the radiology team makes a comprehensive report of the MRI and pre-surgical X-ray images (if available) and if they identify extra/missing ribs or other anatomical exceptions such as lumbarization or sacralization of vertebral body or more/less number of vertebrae, they include that information in their notes and the researchers review these notes before performing the analysis and surgical procedures to ensure accuracy of the analysis for each participant.

### Spinal cord neuroanatomical mapping at the lumbosacral enlargement

**Figure 1:**
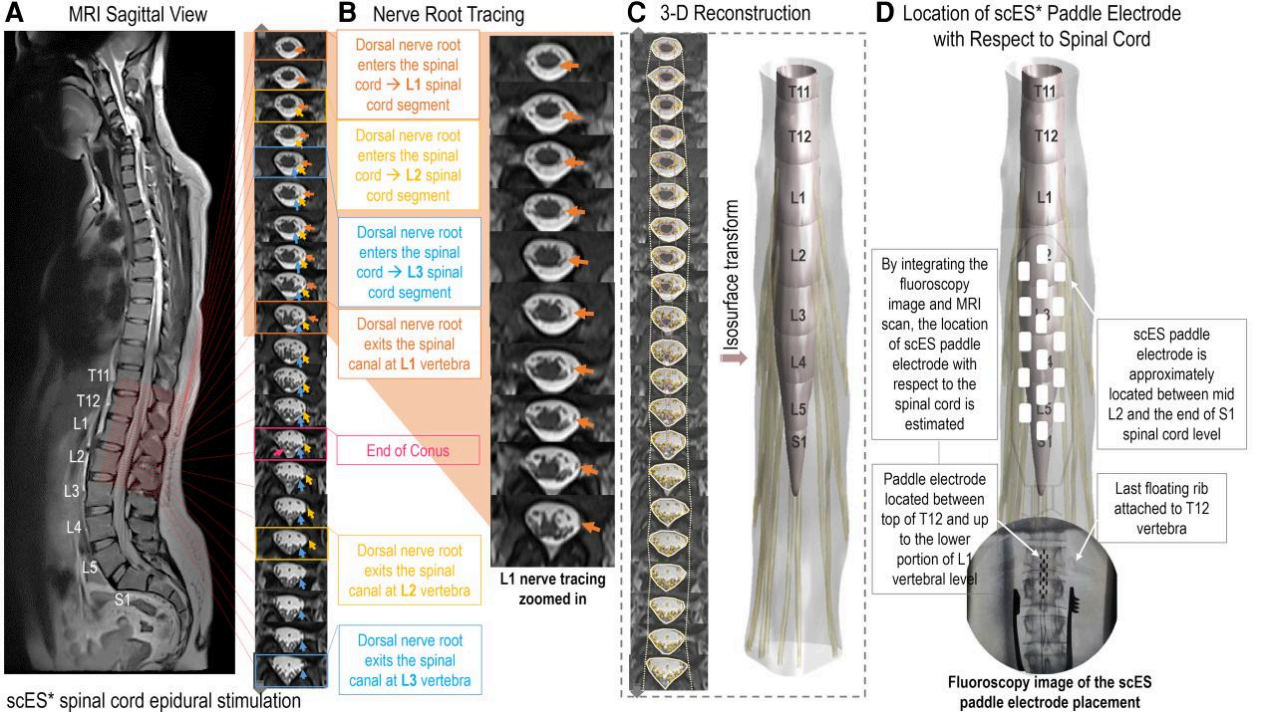
**Neuroanatomical mapping of the spinal cord at LSE.** (**A**) MRI sagittal view of the whole spine in an individual with a cervical spinal cord injury. (**B**) Axial images of the lumbosacral region from high spatial resolution MRI recording, with the arrows pointing at the dorsal nerve roots from the vertebral site of the exit back to the location of entering the spinal cord to show how spinal cord levels are identified using the nerve root tracing technique. (**C**) Process of tracing and labelling the spinal cord region, cerebrospinal canal and nerve roots in each axial image and using the isosurface function to build a reconstructed model of the lumbosacral spinal cord from the segmented images. (**D**) Process of integrating intraoperative fluoroscopy images of the paddle placement with the preoperative MRI recordings in order to estimate the location of the paddle array on the spinal cord model.

Recorded MRI axial scans with high spatial resolution (3 mm slice thickness and zero gap) were used to locate and trace the dorsal and ventral nerve roots that float in the cerebrospinal fluid. As the spinal cord typically ends at the L1 vertebral level, the nerve roots that enter the spinal cord at the LSE start to elongate and exit the spinal canal further distally at the corresponding vertebral levels (L1, L2, …, S1). The spinal cord lumbar segments assigned to L1, L2, …, S1 were anatomically estimated by identifying the set of nerve roots (dorsal and ventral roots on the left and right sides) that exit the spinal canal at each vertebral level and back-tracing those nerve roots into the spinal cord body. The process of nerve root tracing and estimating spinal cord L1–S1 segments is referred to as spinal cord neuroanatomical mapping in this study and was performed manually by an expert analyst. Furthermore, the axial images of the lumbosacral spinal cord were traced and labelled based on the area of the cerebrospinal canal, spinal cord tissue and nerve roots. The expert analyst was trained by the neurosurgery and the radiology teams to accurately read the spinal cord MRI images and perform the segmentation and nerve root tracing. All the images and consequent analysis were reviewed by the research team members for accuracy. The analyst was blinded to the participants’ functional outcomes. A 3D model of the spinal cord of each individual was then reconstructed using custom-written codes in MATLAB. This step can also be performed using available 3D modelling platforms such as Sim4Life, COMSOL Multiphysics, etc. The estimated neuroanatomical levels of spinal cord were visualized on the 3D reconstructed model of the lumbosacral region (Fig. 1A–C). Total length of the spine (from top of v-C1 to the end of v-S5) and spinal cord (top of the cervical level to the end of conus medullaris), length and volume of the spinal cord and cerebrospinal canal at the LSE and the conus area (from top of sc-L1 to the end of conus) as well as the location of the conus tip with respect to the vertebral body were also measured and reported in order to measure the amount of variability across individuals.

### Identifying the location of scES paddle on the spinal cord

After the scES implantation surgery, anterior–posterior and lateral X-ray images of the spinal cord at the location of the scES paddle electrode implant were obtained prior to participant discharge. The anterior–posterior image was used to identify the T12 vertebra based on the location of the last floating rib, while the lateral image was used to identify the exact locations of the rostral and caudal ends of the paddle electrode with respect to the vertebral body. After identifying the exact location of the paddle electrode with respect to the vertebrae using X-ray images, the location of the paddle was estimated with respect to the spinal cord by integrating the lateral X-ray with the sagittal and axial MRI scans. Based on the length of the paddle electrode (46.5 mm for Medtronic Specify® 5-6-5 lead), 15 MRI axial slices (total of 15 × 3 mm = 45 mm in length) that best describe this location were identified. The paddle electrode was placed on the 3D model based on the location of the identified 15 axial slices (Fig. 1D). Percentage of volumetric coverage of lumbosacral spinal cord by scES paddle electrode was calculated for each participant using the methodology explained in Supplementary Fig. 2.

### Neurophysiological spatiotemporal mapping post implant

**Figure 2:**
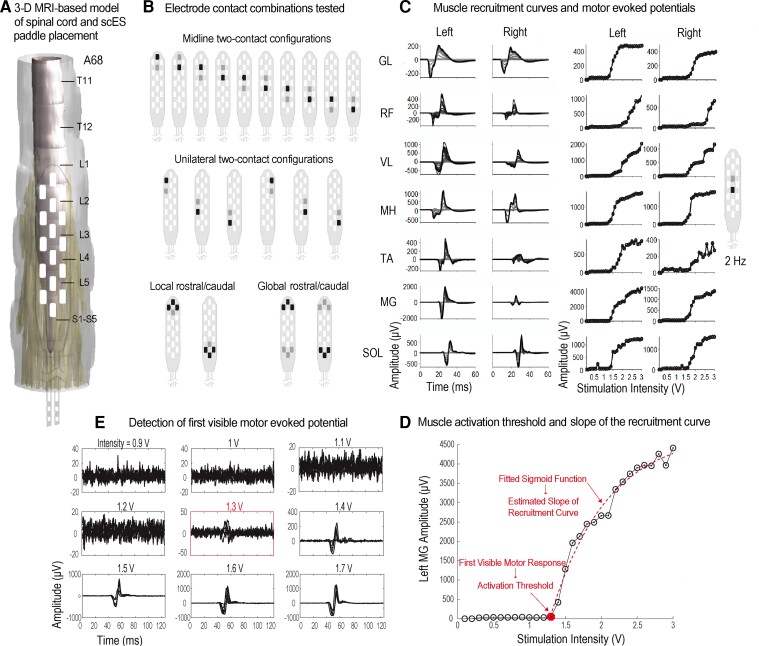
**Neurophysiological mapping post implant.** (**A**) An example of reconstructed spinal cord model and estimated paddle placement (Subject A68). (**B**) List of electrode contact combinations tested during spatiotemporal neurophysiological mapping post implant in supine position (frequency 2 Hz, pulse width 450 or 1000 µs, intensity voltage or milliampere ramps up from zero until all muscles show induced activation). (**C**) Examples of corresponding overlaid motor evoked potentials (overlaid temporal activities with amplitudes low to high correspond to evoked response averages to different stimulation intensities) and muscle recruitment curves (peak-to-peak amplitude µV) for each electrode combination tested across 14 muscles including left and right SOL, MG, TA, VL, RF, MHs and GL. (**D**) Each panel shows overlaid responses to stimulation pulses at the same stimulation intensity (usually between 3 and 5 pulses are delivered for each stimulation intensity) and the example of first visible motor evoked potential during stimulation ramp-up. (**E**) Identifying the activation threshold, i.e. the amount of intensity that led to first visible motor evoked potential and the estimation of the slope of the recruitment curve from fitted sigmoid function.

After the surgical wounds heal (2–3 weeks) and the participant was clinically cleared following implantation surgery, neurophysiological spatiotemporal mapping is conducted with the participants in the supine position. Stimulation parameters for scES were scanned by testing bipolar electrode configurations in various locations of the paddle electrode including midline and unilateral bipolar two electrodes, and global and local bipolar multi-electrode configurations (Fig. 2A and B). In global configurations, the generated electric field is extended across the electrode array between cathodes and anodes and the direction of the field switches from upward to downward depending on the polarity of active electrode contacts. These two configurations impact a larger area of the spinal cord and nerve roots that are covered by the scES electrode paddle. On the contrary, multi-electrode local and two-electrode unilateral and midline configurations generate a more focused electric field that impacts smaller regions of the excitable tissue. During the mapping experiments and for each electrode configuration (total of 20 configurations), stimulation intensity was ramped up until all proximal and distal leg muscles demonstrated stimulation-induced activations. Stimulation frequency was set at 2 Hz and pulse duration was set at either 450 or 1000 µs. Surface electromyogram (EMG) signals were recorded at 2000 Hz using a 24-channel hard-wired AD board and custom-written acquisition software (Labview, National Instruments, Austin, TX, USA). EMGs (MotionLab Systems, Baton Rouge, LA, USA) were recorded from left and right soleus (SOL), medial gastrocnemius (MG), tibialis anterior (TA), vastus lateralis (VL), rectus femoris (RF), medial hamstrings (MHs) and gluteus maximus (GL), a total of 14 muscles (7 on each side). In addition, two surface electrodes were placed over the paraspinal muscles, symmetrically lateral to the epidural electrode array incision site. These two electrodes were used to record the stimulation artefact from the implanted electrode. First visible motor evoked potentials for each muscle during intensity ramp-up were detected manually by an expert observer (Fig. 2E). The corresponding intensity value to the first visible motor evoked potential is referred to as ‘activation threshold’ in this study. The activation threshold unit is either in ampere or voltage depending on what type of scES model the participants are implanted with. Within the group of participants with the same scES model, the maximum stimulation intensity that activated a muscle vary across participants. This variation can be due to various factors such as the differences in the anatomical distances between the site of stimulation and the excitable tissues, the conductivity properties of the tissues and medium between the location of the implant and the nerve roots or the angle and directions of the nerve roots with respect to the electric field generated by scES. In our study, we normalized the activation thresholds within each subject to their own maximum threshold across all electrode configurations tested in order to: (i) remove dependency on voltage versus ampere units and (ii) focus the comparison on the order of the muscles that are getting activated as we ramp-up the stimulation intensity and remove the effects of variabilities due to other factors described above. From the muscle recruitment curve, the slope of the curve during the intensity ramp-up was estimated by fitting a sigmoid function to the ramp-up portion of the curve (Fig. 2C and D). We used the activation threshold (normalized) and the slope of the recruitment curve as the two features from the spatiotemporal mapping data for the rest of the analysis.

### Statistical analysis

Activation threshold and slope values were evaluated with linear mixed model including a random intercept and slopes for configurations (local/global) and direction (rostral/caudal) for each participant. Independent variables in the model were muscle type (GL, MG, MH, RF, SOL, TA and VL), side (left/right), the interaction of configuration and direction and the interaction of muscle, configuration and direction. To evaluate different two-by-two group comparisons, linear contrasts were built on interaction terms. Linear contrasts are two-group comparisons and are evaluated by adjusted *t*-tests. The analyses were performed for all participants and per paddle placement group (L1–L5/S, L2-S and L4-S). Significance level was set to 5% and all *P*-values were adjusted for multiple testing using the Bonferroni correction.^21^ Pearson correlation coefficients were used to measure the correlation between the total spine length, total spinal cord length and lumbosacral spinal cord length and considered significant if their *P*-values were <0.05. Pearson correlation coefficients were also used to perform correlation analysis between the level of the spinal cord that was being stimulated with scES and normalized activation threshold values and the slope of recruitment curves for seven leg muscles. The Pearson correlation coefficient (*r*) values were classified as negligible (0–0.3), low (0.3–0.5), moderate (0.5–0.7), high (0.7–0.9) and very high (0.9–1), based on classification table suggested by Hinkle *et al*.^22^ The analysis of the largest enlargement cord segment was performed using repeated measure ANOVA with *post hoc* tests comparing the largest cord level to all other levels. All tests were two sided. Statistical analyses were performed in SAS 9.4 (SAS Inc., Cary, NC, USA).

## Results

Clinical and demographic information of enrolled participants is provided in Table 1. Participants’ ages ranged from 21.9 to 55.7 (34 ± 10.9 years), with a 5:1 ratio of males to females, and average time since injury was 8.2 ± 9.9 years. Six individuals were classified as AIS A, and six as AIS B. Level of injury spans from C4 to T4 across participants.

**Table 1:** Participants’ clinical and demographic information

Publication ID	Sex	Age (year)	TSI (year)	AIS	Level of injury
A64	M	55.7	38.6	A	C4
A41	M	24.0	7.2	A	C4
A102	F	29.1	4.4	A	C4
A68	M	35.0	3.8	A	C5
A60	M	23.3	3.2	A	T3
A59	M	26.6	2.5	A	T4
B47	M	43.3	8.2	B	C4
B21	M	31.0	6.9	B	C4
B38	M	21.9	3.3	B	C4
B52	F	51.5	8.0	B	C5
B23	M	32.0	3.3	B	C5
B45	M	35.0	9.1	B	C7

AIS, American Spinal Injury Association Impairment Scale; TSI, time since injury.

### Neuroanatomical characteristics of human spinal cord

**Figure 3:**
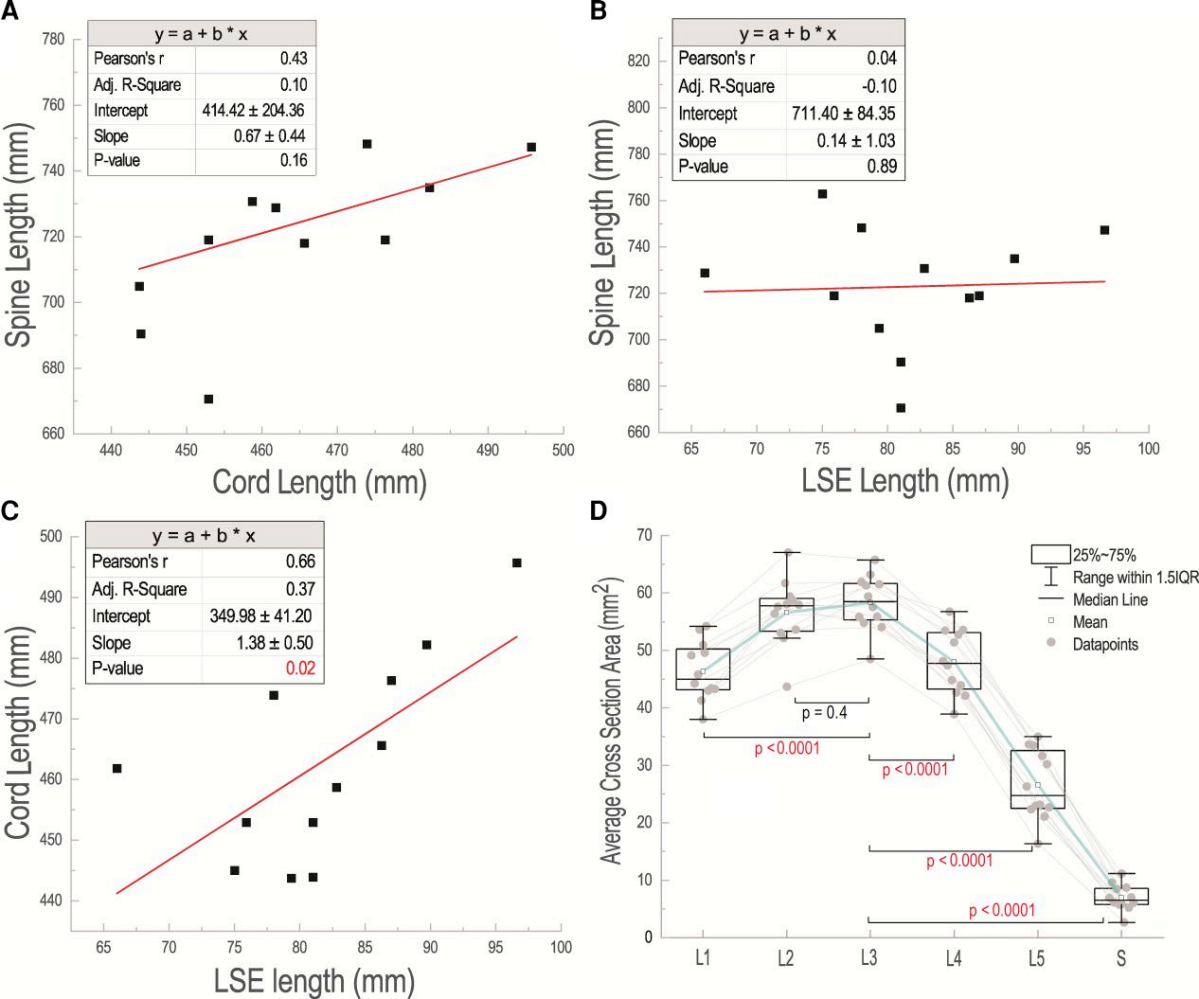
**Neuroanatomical characteristics of the spinal cord in humans.** (**A**) Correlation plot between whole spine length and whole spinal cord length for 12 participants (*n* = 12). (**B**) Correlation plot between whole spine length and LSE and conus length (*n* = 12). (**C**) Correlation plot between whole spinal cord length and LSE (*n* = 12). The linear fitted correlation lines are shown in the plots and the statistical values including Pearson *r*, adjusted *R*-square, intercept, slope and *P*-value are included in the corresponding tables (**A**–**C**). (**D**) Individual values and boxplots of average cross-section areas of the spinal cord at lumbosacral levels L1, L2, …, L5 and sacral (S) region. Repeated-measures ANOVA with *post hoc* tests was used to compare the largest level to all other levels. For L3 to L1 comparison: *t*-value = 5.77; for L3 to L2 comparison: *t*-value = 0.82; for L3 to L4 comparison: *t*-value = 4.99; for L3 to L5 comparison: *t*-value = 15.34; for L3 to S comparison: *t*-value = 24.80.

**Figure 4:**
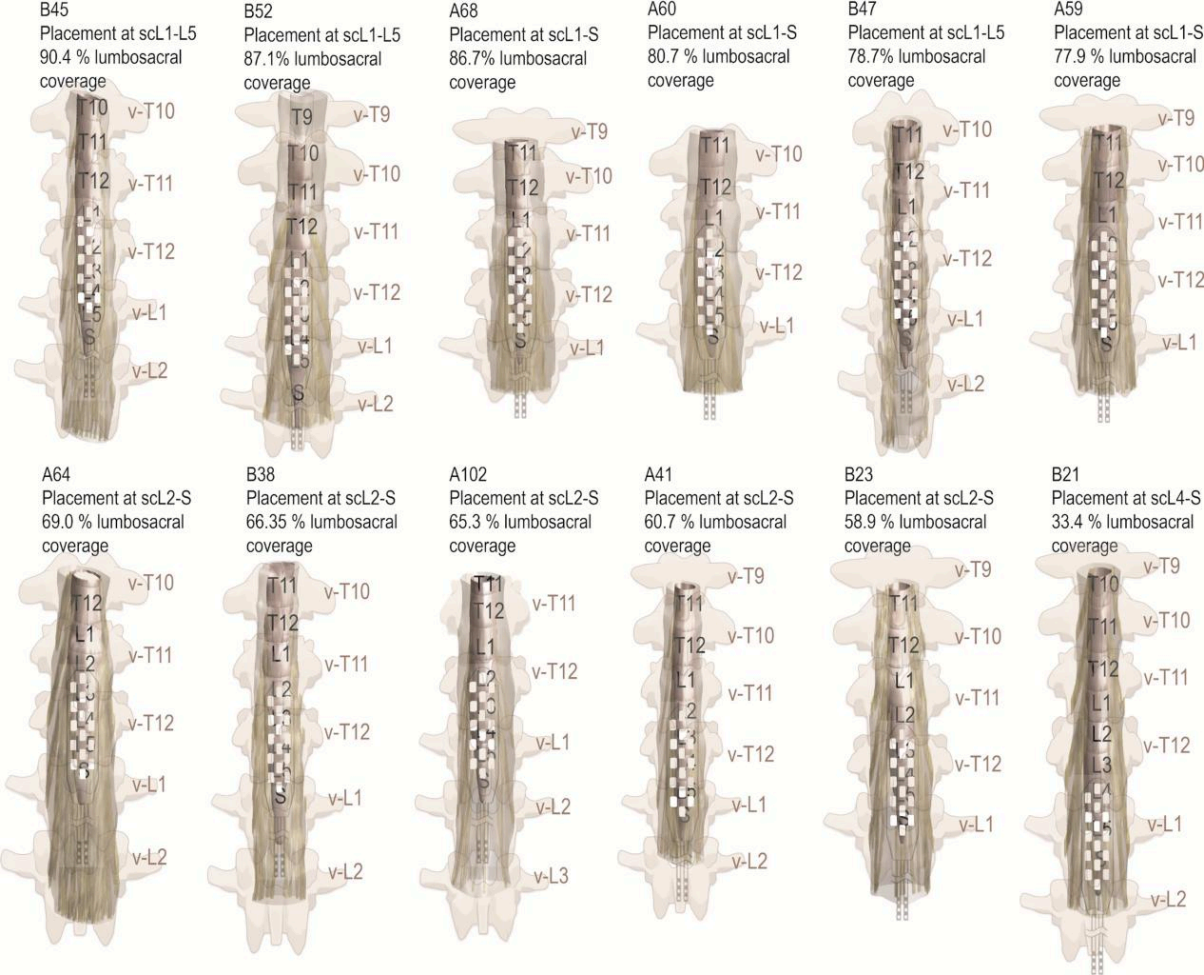
**Three-dimensional MRI-based reconstructed models of the spinal cord at LSE.** Lumbosacral spinal cord reconstruction from high-resolution MRI scans, estimated epidural stimulation paddle placement and drawings of the location of vertebral bodies with respect to spinal cord levels for 12 participants in this study.

The estimated anatomical characteristics of the spinal column and spinal cord segments for each participant based on quantitative MRI analysis are provided in Figs 3 and 4, and Supplementary Table 1. The size (length and volume) and location of the spinal cord segments with respect to the vertebral bodies at LSE are highly variable across individuals (Supplementary Table 1). The estimated location of spinal cord L1 segment (sc-L1) with respect to the vertebrae (v) ranges from v-T10/T11 gap to the end of v-T11 across individuals. Location of sc-L2 ranges from mid-v-T11 to top of v-T12. Location of sc-L3 ranges from end of v-T11 to mid-v-T12. Location of sc-L4 ranges from top of v-T12 to v-T12/L1 gap. Location of sc-L5 ranges from mid-v-T12 to top of v-L1. Location of sc-S1 ranges from end of v-T12 to mid-v-L1. Location of the tip of the conus ranges from top of v-L1 to mid-L2. Figure 3 provides correlations between total length of the spinal column, total length of the spinal cord and length of spinal cord LSE as well as the boxplots of average cross-section areas of the spinal cord at L1, L2, L3, …, S levels. The correlation analysis shows that there are no statistically significant correlations between the total length of the spinal column and total length of the spinal cord or the length of the LSE (Fig. 3A and B). However, there was a significant moderate correlation between total length of the spinal cord and length of the LSE (Fig. 3C). In Fig. 3D, the average values of the cross-section areas of the spinal cord levels L1, L2, L3, …, S for 12 participants are plotted with boxplots and it shows that sc-L3 on average has the largest cross-sectional area (maximal enlargement) on the LSE. Repeated-measures ANOVA with *post hoc* tests was used to compare the largest level to all other levels. This analysis shows that L3 is larger than all other levels (shown by all differences being positive) and all differences are statistically significant except for the difference with L2.

The intraoperative fluoroscopy and postoperative X-ray images were used to identify the location of the paddle electrode with respect to the vertebrae (Fig. 1D). By identifying the segmental locations of the spinal cord in relation to the vertebral bodies (Supplementary Table 1), we estimated the location of the paddle array with respect to the lumbosacral spinal cord. Figure 4 illustrates the reconstructed spinal cord at LSE with identified spinal cord levels via nerve root tracing and scES paddle placement overlaid on the 3D models for each participant in coronal view (*n* = 12). The locations of spinal cord levels and conus tip with respect to the vertebrae are derived from the MRI scans and indicated with the illustrations of the vertebral bodies in these 3D models. These models visualize the amount of variability among individuals regarding the size and position of spinal cord levels with respect to the positioning of the scES implant as well as the location of encircled vertebrae. Volumetric coverage of the lumbosacral spinal cord ranges from 33.4 to 90.4% across participants.

### Links between the neuroanatomical and neurophysiological maps

**Figure 5:**
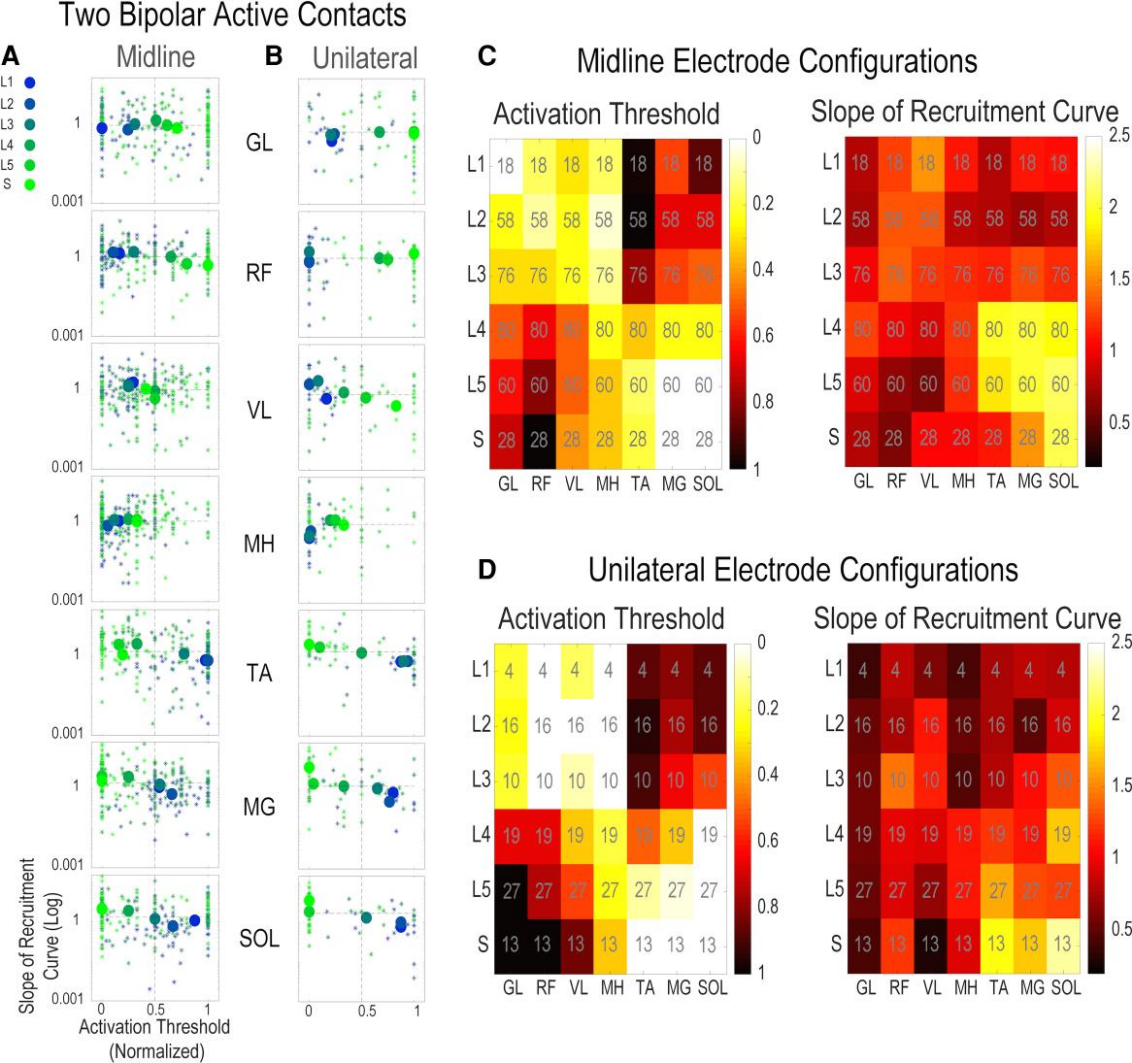
**Relation between neuroanatomical mapping of lumbosacral and neurophysiological muscle responses.** (**A**) Scatter plots for the slope versus activation threshold values across seven muscles (left and right muscles combined) for midline two bipolar contact electrodes. The scale is corresponding to the stimulation site at L1, L2, …, L5 and S spinal cord levels. (**B**) Scatter plots for the slope versus activation threshold values across seven muscles for unilateral two bipolar contact electrodes. (**C**) Heatmap plots for activation threshold values and slope values across seven muscles and L1, L2, …, L5 and S spinal cord levels for midline two bipolar contact electrodes. (**D**) Heatmap plots for the average of normalized activation threshold and slope values across seven muscles and L1, L2, …, L5 and S spinal cord levels for midline two bipolar contact electrodes. The Pearson correlation coefficients for comparing the targeted spinal cord levels by the activated electrode contacts (both midline and unilateral) with the normalized activation threshold values showed negative correlations for upper leg muscles: GL, RF, VL and MH and positive correlations for lower leg muscles: MG, TA and SOL with all the correlation values being statistically significant (*P* < 0.0001). For midline electrode configurations, the correlation results between the slope of the recruitment curves and spinal cord levels were not statistically significant for GL (*P* = 0.08) and MH (*P* = 0.09) but there were significant and positive correlations for VL (*P* < 0.0001) and RF (*P* < 0.0001) and there were significant and negative correlations for MG (*P* < 0.0001), TA (*P* = 0.02) and SOL (*P* < 0.0001). For unilateral electrode configurations, the correlation results between the slope of the recruitment curves and spinal cord levels were not statistically significant for GL (*P* = 0.3), RF (*P* = 0.1) and MH (*P* = 0.7) but there were significant and positive correlations for VL (*P* = 0.0008) and there were significant and negative correlations for MG (*P* = 0.01), TA (*P* = 0.001) and SOL (*P* = 0.03).

The patterns of muscle activations, based on activation threshold and the slope of recruitment curves across all individuals, are provided in Fig. 5. The midline versus the unilateral configurations (indicated in Fig. 2B) were tested and the location of the stimulation (cathode electrode/−) with relation to the spinal cord levels were identified. The individual data points of activation thresholds plotted against the estimated slope values grouped by gradient colours (blue to green) based on the targeted spinal cord levels and the median values are represented with larger circle markets in Fig. 5A and B. In the scatter plots shown in Fig. 5A and B, one can follow the light green marker (indicating the sacral region of the spinal cord being the target of the simulation) across the proximal and distal muscles and observe that the light green marker swings from the far-right to the far-left side indicating lower sensitivity (higher activation threshold) of the spinal cord sacral region for activating proximal muscles and higher sensitivity (lower activation threshold) for the distal muscles. Similar pattern exists for the dark blue marker (sc-L1) but in the opposite direction. This trend is also observable in the activation threshold heatmap plots on Fig. 5C and D. This shows that moving down the lumbosacral spinal cord levels (L1, L2, …, S), a reciprocal pattern of activation occurs at around sc-L4, where proximal muscles are activated first and distal muscles are activated later (while the stimulation intensity was ramped up) when active electrode contacts are targeting sc-L1, sc-L2 and sc-L3, and the pattern changes to distal muscles activated first and proximal muscles activated later when targeting sc-L4, sc-L5 and the spinal cord sacral region. Among the seven leg muscles that were the focus of this study, MH has the least sensitivity to the level of lumbosacral spinal cord that is the target of stimulation and this muscle mostly shows early induced activation and less selectivity compared with other muscles at L1 to S spinal cord levels. The effects of the slope are less prominent compare with the activation threshold in Fig. 5A and B; however, there is a slight trend from sharper slopes on the left side of the scatter plots to slower slopes on the right side of the plots across spinal cord levels (blue to green markers) for RF (midline configurations), VL (unilateral configurations) and TA, MG, and SOL (both midline and unilateral configurations). This indicates that when the activation threshold is high (i.e. less sensitivity), the slope of the recruitment curve is also typically flatter and vice versa.

The Pearson correlation coefficient analysis for the data presented in Fig. 5 shows that there were statistically significant correlations between the spinal cord levels targeted by stimulating midline or unilateral electrode contacts and the normalized activation thresholds across all muscles. The correlation coefficients were negative for upper leg muscles, i.e. GL, RF, VL and MH, and the coefficients were positive for lower leg muscles, i.e. MG, TA and SOL, which confirms the reciprocal pattern of activation described previously. For correlation analysis of spinal cord levels and slope of recruitment curve values, there are no statistically significant correlations between GL and RF and spinal cord levels when stimulated at midline, and no significant correlations between GL, RF and MH and spinal cord levels when stimulated unilaterally. For all the other muscles, the correlation coefficients were statistically significant with positive correlations between upper leg muscles and spinal cord levels and negative correlations between lower leg muscles and spinal cord levels. The summary of the correlation analysis is presented in Supplementary Table 3.

**Figure 6:**
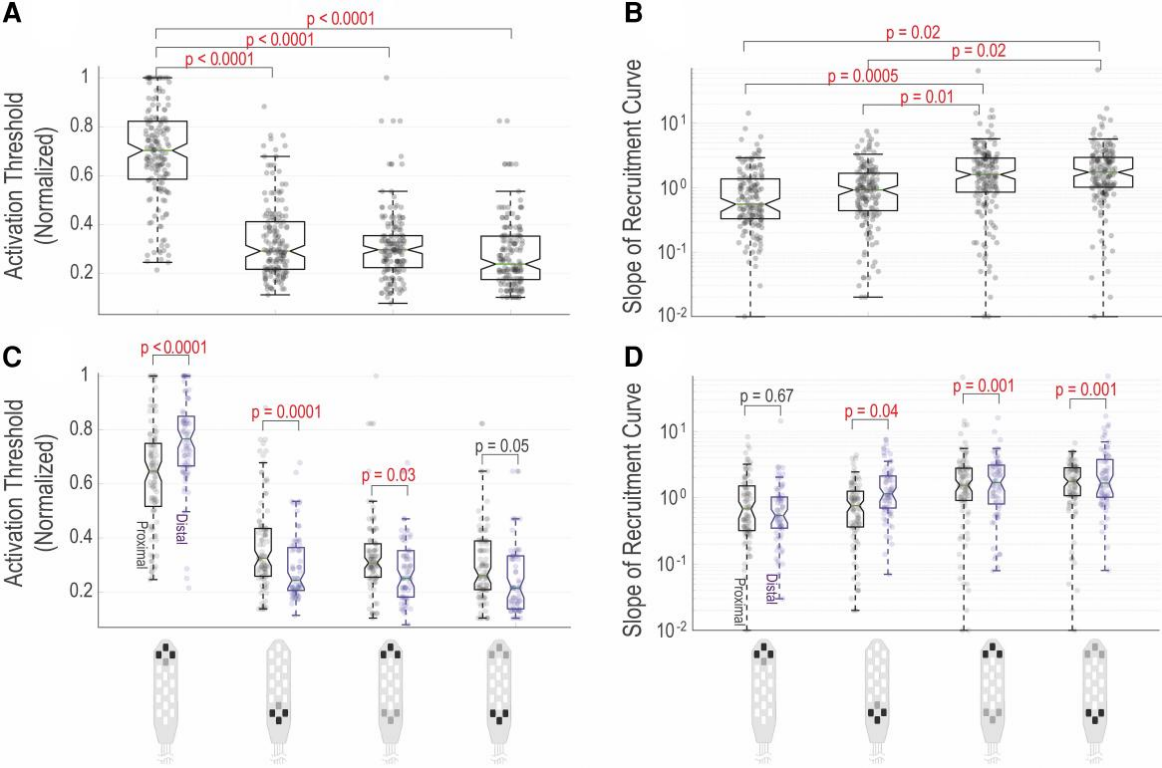
**Narrow and wide field multi-electrode configurations.** (**A**) Activation threshold values across 12 participants and 14 leg muscles (7 muscles on each side). (**B**) Slope of recruitment curve values across 12 participants and 14 leg muscles. (**C**) Activation threshold values across 12 participants, separated by proximal (GL, RF, VL, MH) and distal (SOL, MG, TA) muscles. (**D**) Slope of recruitment curve values across 12 participants, separated by proximal and distal muscles. *P*-values were obtained from linear contrasts built on an interaction term in a mixed linear model. This model includes a random intercept and slopes for configuration (local/global) and direction (rostral/caudal) for each participant. Independent variables in the model were muscle type (GL, MG, MH, RF, SOL, TA, VL), side (left/right), the interaction of process and direction and the interaction of muscle, configuration and direction. The linear contrasts, comparing two-by-two factor levels, were statistically evaluated with model-adjusted *t*-tests. The values of these tests as well as the degree of freedom for the overall model are included in the Supplementary material.

The boxplots in Fig. 6 show the statistical differences between the muscles activation patterns when global versus local and rostral versus caudal stimulation configurations were used. Complete list of statistical analyses on this data is presented in Supplementary Table 2. The results show that local caudal configuration has a significantly lower activation threshold than local rostral configuration; however, there is no difference in activation threshold values between local caudal and global (rostral and caudal) configurations (Fig. 6A). On the other hand, the slope of recruitment curve values indicates significant differences between local configurations (rostral and caudal) and global configurations (rostral and caudal) with global configurations showing greater slope (faster rise in the recruitment curve) on average (Fig. 6B).

These findings show that for global configurations, there is no significant difference for either activation threshold or slope when cathodes are flipped from rostral to caudal. This suggests that (i) the electric field generated by the global configurations are likely impacting large number of dorsal (at lower stimulation intensities) and ventral (at higher stimulation intensities) nerve roots at their entry zones to the spinal cord levels at LSE which leads to lower activation thresholds and higher slopes across all muscles; and (ii) from muscle recruitment standpoint, when stimulating nerve rootlets at entry zones with global configurations, the location of cathodes or the vertical direction of the electric field may not play a significant role.^23,24^ When stimulating caudally using local configurations, the induced electric field is likely targeting multiple elongated dorsal and ventral nerve roots far away from their entry zones to the spinal cord levels therefore leading to less selectivity of the activated muscles (similar to global configurations) but lower slopes of recruitment curves, possibly due to the number and/or types of activated nerve fibres and interneurons.

The breakdown of activation threshold and slope values between proximal and distal muscles and the statistical comparison show that the proximal muscles have a lower threshold when a local rostral configuration was used compared with distal muscles (Fig. 6C and D). However, for the local caudal and global rostral, this trend switches to distal muscles having lower activation thresholds. For the slope values, there is no significant difference between proximal and distal muscles when stimulating locally at proximal location, but there are significant differences between proximal and distal muscles for local caudal configuration and global configurations with distal muscles showing, on average, a faster rise on the recruitment curve.

### Preoperative paddle placement prediction for scES implantation

**Figure 7:**
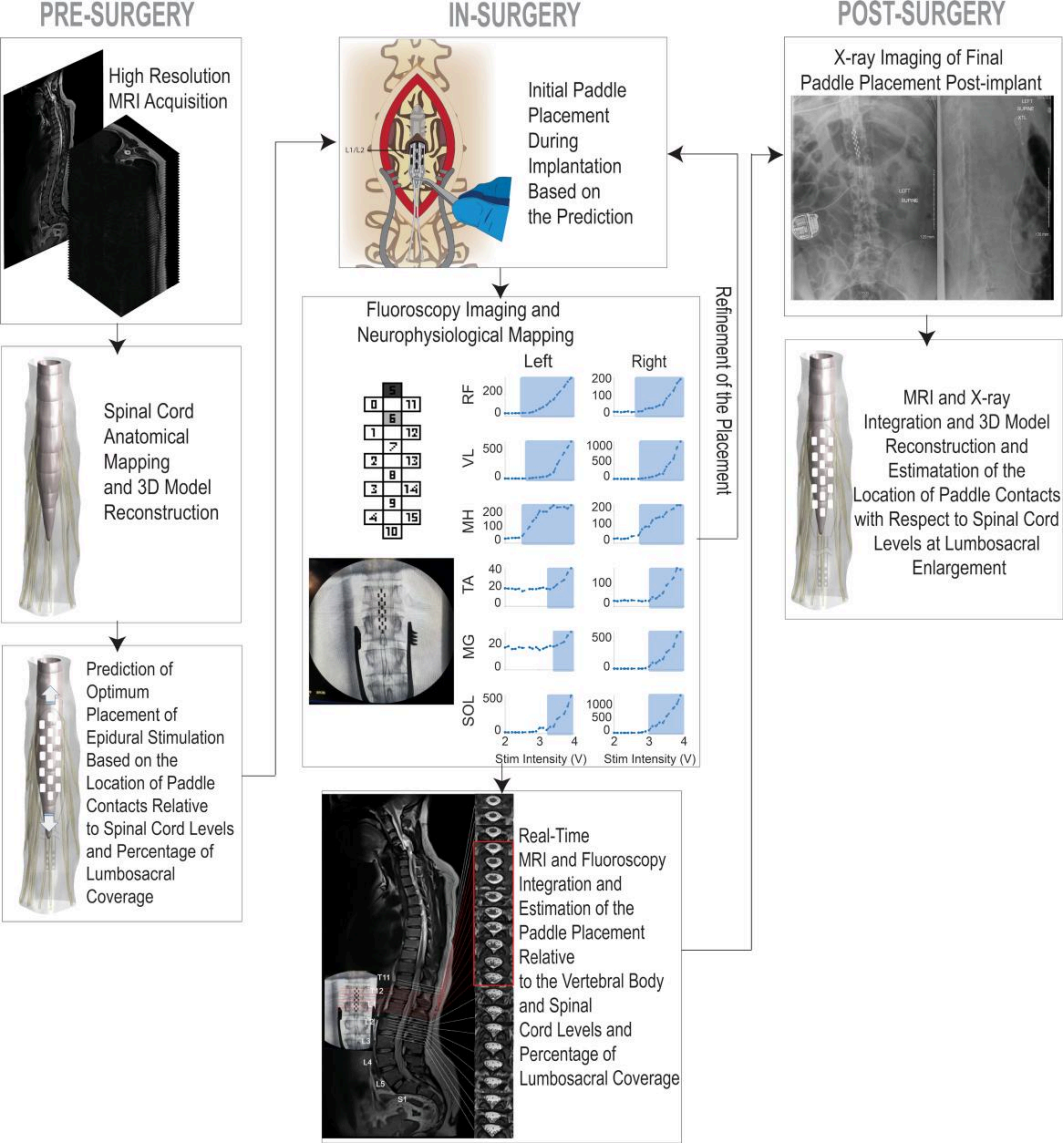
Proposed spinal cord epidural stimulation implant surgical protocol.

The development of individual-specific spinal cord neuroanatomical mapping and 3D reconstruction modelling of the LSE enables prediction of the optimum placement for each participant prior to the scES implantation surgery. Upon future validation, we expect that this protocol will (i) identification of the anatomically optimal location for laminotomy for each participant, (ii) time reduction in intraoperative search for finding the best placement and (iii) identification of the conus tip to ensure full coverage of the spinal cord tissue thereby avoiding placement below the conus. The proposed scES surgical implantation protocol is summarized in Fig. 7.

## Discussion

In this study, we have provided a quantitative neuroimaging methodology and an in-depth analysis of the neuroanatomical characteristics of the human spinal cord using high-resolution MRI scans in order to examine the amount of variability that exists among individuals with chronic SCI and whether this variability can influence the scES implantation procedure. We linked the outcomes of the neuroanatomical mapping generated from high-resolution scans with the neurophysiological spatiotemporal mapping performed post-scES implant with participants laying supine with no intention to initiate leg movements and stimulation intensity gradually increasing while the frequency was fixed at 2 Hz.

The analysis of the neuroanatomical characteristics of the spinal cord provided in this study indicates that there is a considerable amount of variability across individuals regarding the volume and length of the spinal cord and of the enclosing vertebrae. The location of the end of the conus with respect to the vertebrae varies across individuals (ranges from top of v-L1 to mid-v-L2). Similarly, variability exists among participants in the sc-L1, L2, …, S levels of lumbosacral region of the spinal cord with respect to surrounding vertebrae (Figs 3 and 4 and Supplementary Table 1). The findings also suggest that by only measuring the length of the spinal column in individuals, the length of the LSE cannot be estimated and having a longer (or shorter) spinal column does not necessarily indicate a longer (or shorter) spinal cord (Fig. 3A). The average values of the cross-section areas of the spinal cord at sc-L1, L2, …, S levels across individuals (Fig. 3D, *n* = 12) match the findings of a recent cadaver study^24^ (*n* = 9), showing sc-L3 has the largest spinal cord transverse diameter (directly related to the cross-section area) and indicates the area of maximal size of the LSE. These findings also show that the length of spinal cord from the top of LSE (sc-L1) to the end of conus in all individuals ranges from 66.0 to 96.6 mm which is greater than the length of the current paddle electrode design (Medtronic Specify® 5-6-5 lead), 46.5 mm, thereby requiring compromise during surgical implantation to provide coverage of the spinal cord segments that are most essential for the individual and their most significant neurological deficits that need to be addressed with epidural stimulation.

Neurophysiological mapping of the human spinal cord to identify the topographical recruitment patterns of the LSE that target activation of leg muscles has been performed previously using various experimental methodologies.^23,25–28^ Several studies have used epidural stimulation in humans to induce motor responses in leg muscles and identify the patterns of motoneuron pool activation in relation to the spinal cord levels and afferent and efferent nerve roots.^23,27^ In this study, we demonstrated the effects of different active electrode combinations (bipolar midline, bipolar unilateral, multipolar local and global with cathodes location rostral and caudal) in the muscle evoked responses. Particularly in Fig. 5, when comparing the activation threshold values for midline versus unilateral configurations (in both scatter plots and the heatmap plots), there is a trend towards higher selectivity when active electrodes are selected unilaterally compared with those in the midline. This observation is in agreement with previous claims demonstrating that stimulation targeting the dorsal roots entry zones to the spinal cord can lead to better neuromodulation outcomes.^17,24^ However, these results should be interpreted carefully, as there were a greater number of midline configurations (10 configurations) that were tested in this study compared with the unilateral configurations (6 configurations). Additionally, in Fig. 6, the results of statistical analysis across local and global configurations (for all muscles and all subjects combined) showed that global configurations have more generalized and less selective effects on the motor pool activations with lower activation thresholds and sharper slopes on average. The results of the local configurations also showed that if stimulated caudally, the selectivity of motoneuronal recruitment is poor, likely due to the close proximity of the high volume of dorsal and ventral nerve roots near the termination part of the conus. However, the slope of the recruitment curves on average are flatter compared with the global configurations. The slope of the recruitment curve (also called the maximum rate of recruitment in other publications^29^) indicates the rate of recruitment of the neuronal fibres as the stimulation amplitude is increased. A sharp slope suggests that the motoneuron pool is highly sensitive to the stimulation versus a flat slope which indicates lower sensitivity or fewer number of motoneurons responding to the stimulation. These findings may also indicate the difference between stimulating the dorsal roots versus stimulating the nerve rootlets at the entry zone to the spinal cord.

Variability in size (length, area and volume) of the lumbosacral spinal cord levels across individuals may result in differing mechanisms of action of scES in neuromodulation interventions among participants. Similar findings were previously presented in studies related to the use of spinal cord stimulation for pain.^30,31^ The same electrode combinations may enable different neurophysiological outcomes across individuals due to targeting different spinal cord regions and activating various spinal cord networks. It should also be noted that other sources, such as the extent, severity and mechanism of the injury, number of residual fibres as well as clinical and demographic factors may also influence the neuromodulatory effects of spinal cord stimulation. Although maximizing coverage of the lumbosacral is necessary, it is not a sufficient criterion to ensure full restoration of function after SCI.

Previous surgical implantation guidelines for epidural stimulation do not highlight the importance of pre-implant MRI-based preplanning approach.^32^ The current surgical protocols for scES implantation rely mostly on the neurophysiological recordings of muscle recruitment patterns during intraoperative stimulation and the placement of the paddle electrode is adjusted until an optimum response is met. However, not all possible paddle placements can be tested in the surgery and therefore an observed optimal response can only be relative to the limited number of placements that were tested and it may only be a local optimum in the search space and not the best possible placement.

In this study, we proposed a modified protocol for scES implantation surgery that uses the high-resolution imaging and preoperative planning for optimum paddle placement to improve the implantation procedure and ensure maximized coverage. Optimizing the paddle placement can also reduce the time in surgery for adjustments and intraoperative neurophysiological assessments which can potentially reduce complications such as placements below conus, risk of infection and risks of repeated surgeries. An accurate knowledge of the location of the implant with respect to the spinal cord levels will also provide a vital tool for the researchers during post-implant mapping and stimulation-based training sessions that allow them to select the electrode combinations and intensities effectively based on an individual’s spinal cord characteristics and allowing rigorous comparisons across individuals and studies which promote understanding of the mechanism of action for modulating multiple systems.^33^

The findings of this study can also help researchers to narrow the search space and shorten the mapping time when looking for optimum configurations for specific motor tasks. However, based on our experience, a subset of neurophysiological mapping should still be performed for each individual due to the unique characteristics of every person’s neuroanatomical response to the stimulation and the inherent variability across subjects. The spinal cord 3D models and the link between the spinal cord segments and the vertebral body can also be useful in the spinal cord transcutaneous stimulation (scTS) paradigm to target proper neural structures both for using scTS as a therapeutic intervention and for determining who might be a good candidate for implantation with scES. Due to its non-invasive design, scTS may reach a broader population of individuals with SCI in the future.

With technology advancements towards newer epidural stimulation models, full-body MRI compatibility is now available post scES implant, and spinal cord neuroimaging allows a follow-up monitoring of the neuroanatomical characteristics and any possible changes that may follow. Additionally, studying the patterns and direction of electric field distribution generated by active electrodes and corresponding activation patterns of excitable tissues based on the MRI-based personalized models of spinal cord can add more clarity to understanding neuromodulation mechanisms of scES.^5,30,34^ Our future studies will focus on using finite-element and mathematical modelling of neuronal dynamics on the personalized 3D models that we introduced in this study.

This study proposes an image-based neuroanatomical mapping approach for spinal cord stimulation and highlights the importance of individual-specific spinal cord modelling for neuromodulation applications in persons with chronic SCI. The amount of variability that exists across individuals regarding the size of the spinal cord and location and direction of the excitable tissue with respect to the stimulation site should be considered in spinal cord neuromodulation studies, and the findings presented in this work should be verified in future studies with larger sample sizes. This study suggests that the amount and sources of variations of the neuroanatomical characteristics of the spinal cord in humans should also be considered when designing the next generation of spinal cord stimulators in order to insure best coverage for all individuals.

## Supplementary Material

fcac330_Supplementary_Data

## Data Availability

All data used in this study will be available upon request. All the algorithms and mathematical formulas used in this study are available within the manuscript or in the Supplementary material.

## Abbreviations

AIS =: American Spinal Injury Association Impairment Scale
AQ_matrix =: acquisition matrix
ASIA =: American Spinal Injury Association
GL =: gluteus maximus
L =: lumbar
LSE =: lumbosacral enlargement
MH =: medial hamstring
RF =: rectus femoris
sc =: spinal cord
scES =: spinal cord epidural stimulation
SCI =: spinal cord injury
SOL =: soleus
TA =: tibialis anterior
VL =: vastus lateralis
