# Supplementary material for: Neuroanatomical mapping of the lumbosacral spinal cord in individuals with chronic spinal cord injury
Source: Brain Commun. 2022 Dec 19;5(1):fcac330. doi: 10.1093/braincomms/fcac330 (PMC9825531; doi:10.1093/braincomms/fcac330)
Supplement: fcac330_Supplementary_Data [file fcac330_supplementary_data.pdf]

Supplementary materials

Step-by-step guide for manual nerve root tracing and prediction placement from MRI

Step1: identify the link between the sagittal and location of each axial image in the lumbosacral region (dashed red lines on the sagittal image in Supp. Fig. 1); **Step 2:** Label each axial image from top to bottom with a number (1-50); **Step 3:** Label each axial image with the corresponding spinal vertebra or the inter-spinal disk space (shown in black text on top of each axial image in Supp. Fig. 1); **Step 4:** Identify and mark the axial slice that shows the end of conus medullaris. Starting from the last axial slice, identify the pairs of nerve roots that are leaving the spinal canal (in Supp. Fig. 1 example the nerve roots are exiting at L3 vertebra), visually trace the nerves back to where they enter the spinal cord tissue. Label the axial image where the nerves entered the cord (shown in red text at the bottom of each slice in Supp. Fig. 1); **Step 5:** Repeat step 4 for every vertebral level (in this example: L2, L1, T12, T11) that are visible in the axial images and trace the nerves from their exit points to their entry points to the spinal cord and label the axial slices with the corresponding spinal cord levels accordingly; **Step 6:** For tracing the L4 nerve root pairs, go back to the last slice and identify the second most lateral nerve roots that are exiting the cerebrospinal space and trace them backwards to the spinal cord. Same method could be used for L5 and S1 levels depending on the quality of the images. **Note**: identifying the exact entry point for L4, L5 and S1 nerve roots into the cord may not be as clear as L1, L2, L3 and higher levels because close to the termination of conus medullaris there is a cluster of nerve connections in very close proximity. Observing the images of spinal cord in cadavers is helpful to perform this task more accurately; **Step 7:** Identify 15 axial slices (this number is selected for axial images with 3 mm slice thickness and zero gap and the 5-6-5 Medtronic electrode paddle size) that would provide the best coverage for lumbosacral spinal cord segments using the 5-6-5 electrode paddle. In our application, we would like partial sacral coverage of the spinal cord and as much as possible coverage for the lumbar region of the spinal cord and therefore in Supp. Fig. 1 example, slices 15 to 30 are marked both on the axial and sagittal images (orange text) to be the predicted optimum placement which corresponds to T12 to L1 vertebrae; **Step 8:** Calculating the volumetric percent coverage of the lumbosacral spinal cord using the formula described in Supp. Fig. 2.

Note: It should be considered that identifying the spinal cord levels with this method is an estimation and it is subject to some errors. It is at the discretion of the surgical team if these estimations are reliable for implementation.

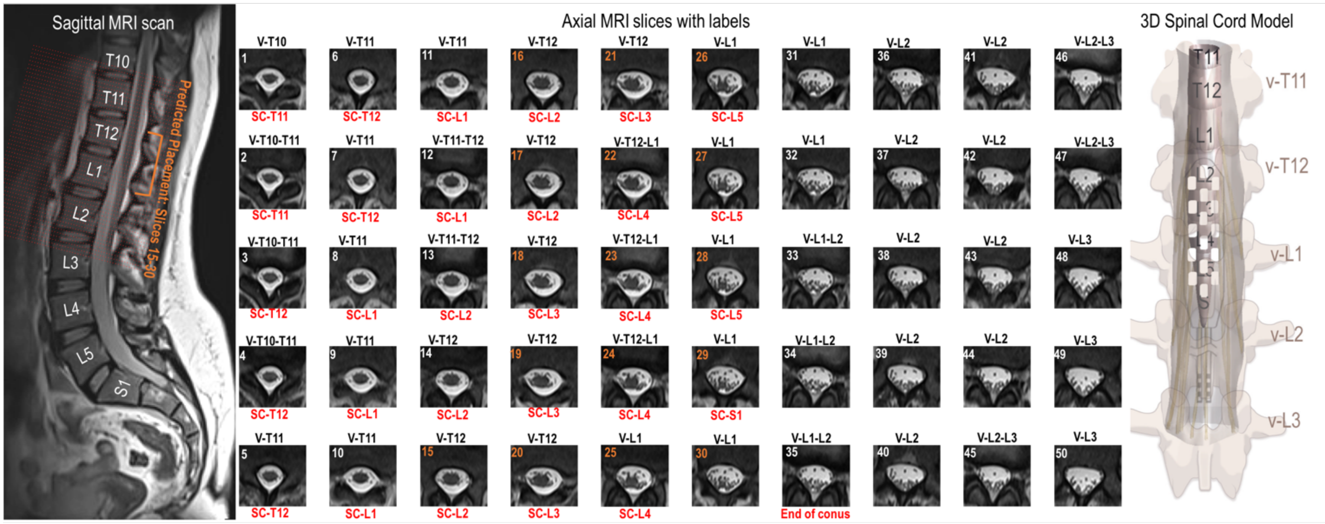

Supplementary Fig. 1 Step-by-step guide to manual spinal cord nerve root tracing and prediction placement from MRI scans.

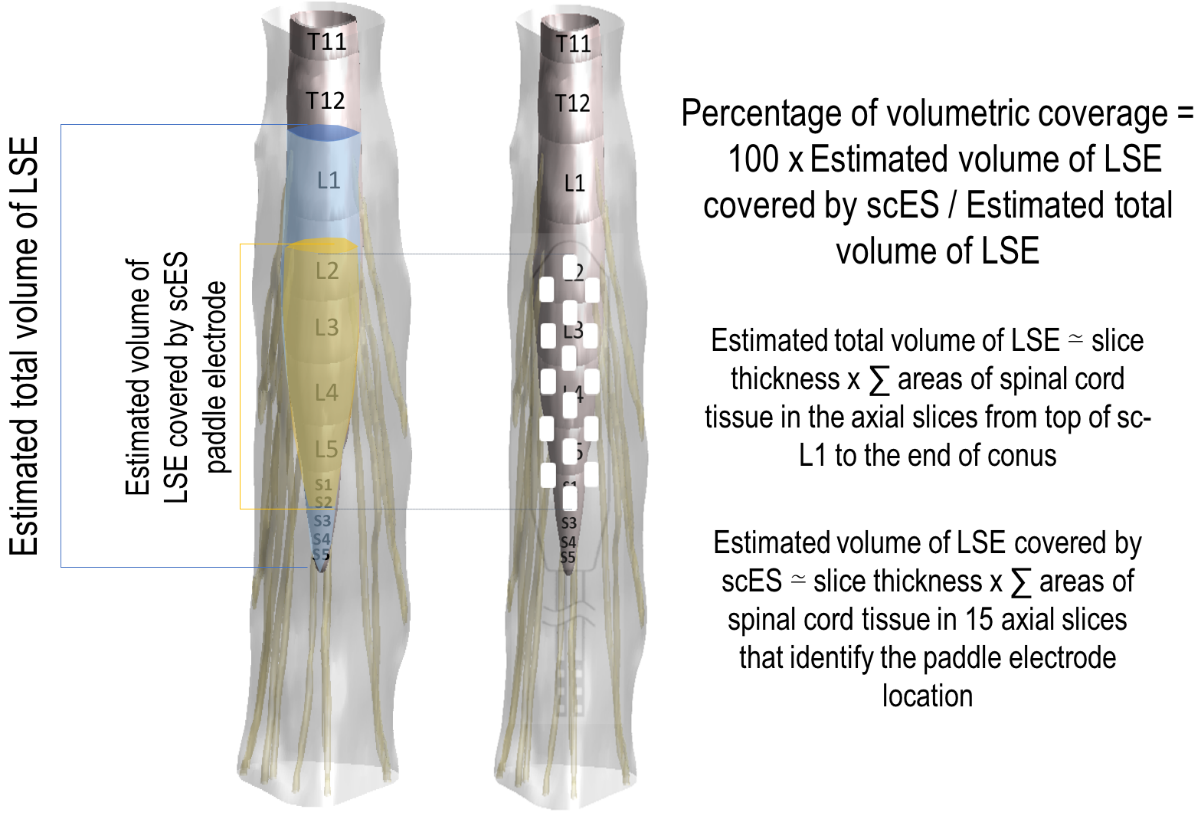

Supplementary Fig. 2 Visualization of the calculation of percentage of volumetric coverage of lumbosacral enlargement (LSE).

Supplementary Table 1 neuroanatomical characteristics of human spinal cord derived from high-resolution MRI recordings of 12 individuals with chronic spinal cord injury. (LSE: Lumbosacral Enlargement; CSA: Cross Section Area; CSF: Cerebrospinal Fluid; Total spine length: total length of the vertebrae and disc space from C1 to S5)

ID	Cord segment	Corresponding Vertebral level	Average Cord CSA (mm^2)	Average CSF CSA(mm^2)	Cord Volume (mm^3)	CSF Volume (mm^3)	Cord segment length (mm)	Total Cord length at LSE (mm)	Total Cord length (mm)	Total spine length (mm)	Total Volume of Cord at LSE (mm^3)	Total Volume of CSF At LSE (mm^3)
B45	L1	T11 (8/8)-T11/T12 (3/3)	49.5	126.4	594.0	1516.9	12.0	66.0	461.8	728.8	2508.4	22071.5
	L2	T12 (1/8)-T12 (4/8)	58.0	175.3	696.0	2103.1	12.0					
	L3	T12 (5/8)-T12 (7/8)	57.5	172.6	517.2	1553.2	9.0					
	L4	T12 (8/8)-T12/L1 (2/3)	42.6	174.7	383.8	1572.1	9.0					
	L5	T12/L1 (3/3) - L1 (2/9)	23.1	226.0	208.0	2033.8	9.0					
	S	L1 (3/9)	5.8	262.6	104.6	4727.6	15.0					
	Conus tip	L1 (8/9)										
B47	L1	T11 (5/8)-T11/T12 (1/2)	41.3	162.5	618.8	2437.9	15.0	78.0	473.9	748.2	2842.8	27877.4
	L2	T11/T12 (2/2)-T12 (4/9)	52.1	168.8	781.3	2531.7	15.0					
	L3	T12 (5/9)-T12 (8/9)	59.4	209.8	712.3	2518.0	12.0					
	L4	T12 (9/9)-T12/L1 (2/3)	44.8	224.9	403.0	2024.5	9.0					
	L5	T12/L1 (3/3) - L1 (2/9)	22.9	255.2	206.5	2296.7	9.0					
	S	L1 (3/9)	5.8	273.9	120.9	5752.0	18.0					
	Conus tip	L1 (9/9)										
B52	L1	T11 (7/7)-T12 (1/7)	37.9	171.1	455.3	2053.4	12.0	81.0	452.9	670.6	2287.0	19480.9
	L2	T12 (2/8)-T12 (5/8)	43.6	210.4	523.6	2525.1	12.0					
	L3	T12 (6/7)-T12/L1 (2/3)	48.4	206.9	581.4	2483.2	12.0					
	L4	T12/L1 (3/3)-L1 (3/7)	38.9	234.0	466.5	2808.0	12.0					
	L5	L1 (4/7) - L1 (7/7)	16.4	269.4	196.5	3232.5	12.0					
	S	L1/L2 (1/3)	2.7	265.8	63.8	6378.7	21.0					
	Conus tip	L2 (5/8)										
A59	L1	T10/T11 (1/2)-T11 (3/7)	50.9	118.4	878.4	2042.1	17.3	86.3	465.6	718.0	3667.5	18284.8
	L2	T11 (4/7)-T11 (7/7)	59.4	137.5	819.1	1897.1	13.8					
	L3	T11/T12 (1/1)-T12 (3/7)	63.2	162.3	872.0	2239.5	13.8					
	L4	T12 (4/7)-T12 (6/7)	51.3	179.8	531.3	1861.3	10.4					
	L5	T12 (7/7) - T12/L1 (2/3)	34.9	204.8	361.6	2119.2	10.4					
	S	T12/L1 (3/3)	8.5	238.2	205.1	5753.5	20.7					
	Conus tip	L1 (6/7)										
A60	L1	T11 (1/6)-T11 (5/6)	43.0	206.3	741.1	3558.4	17.3	75.9	452.9	719.0	3189.5	23400.0
	L2	T11 (6/6)-T12 (1/8)	53.0	196.3	731.3	2709.0	13.8					
	L3	T12 (2/8)-T12 (4/8)	61.3	220.2	634.1	2278.7	10.4					
	L4	T12 (5/8)-T12 (7/8)	56.7	222.6	586.7	2303.5	10.4					
	L5	T12 (8/8) - T12/L1 (2/2)	33.5	231.5	346.2	2395.6	10.4					
	S	L1 (1/8)	8.7	280.8	150.1	4843.6	13.8					
	Conus tip	L1 (5/8)										
B23	L1	T10/T11 (1/2)-T11 (3/7)	45.7	178.3	788.4	3074.8	17.3	82.8	458.7	730.7	3166.9	32891.7
	L2	T11 (4/7)-T11 (7/7)	58.6	223.7	808.9	3086.8	13.8					
	L3	T11/T12 (1/2)-T12 (2/8)	55.8	225.4	770.1	3110.6	13.8					
	L4	T12 (3/8)-T12 (5/8)	43.8	297.1	453.7	3074.8	10.4					
	L5	T12 (6/8) - T12 (8/8)	21.1	332.0	218.3	3436.4	10.4					
	S1	T12/L1 (1/3)	6.2	326.7	127.5	6762.0	17.3					
	Conus tip	L1 (3/8)										
A68	L1	T10/T11 (1/2)-T11 (2/7)	43.4	137.2	598.7	1892.8	13.8	79.3	443.7	704.9	2807.0	21002.7
	L2	T11 (3/7)-T11 (6/7)	57.6	156.4	794.4	2158.9	13.8					
	L3	T11 (7/7)-T12 (1/8)	54.8	170.4	566.7	1764.0	10.3					
	L4	T12 (2/8)-T12 (4/8)	47.3	199.1	489.9	2060.8	10.3					
	L5	T12 (5/8) - T12 (7/8)	22.3	237.8	230.7	2461.6	10.3					
	S	T12 (8/8)	5.2	262.2	126.6	6332.6	20.7					
	Conus tip	L1 (3/7)										
	L1	T10/T11 (2/2)-T11 (3/8)	53.6	125.9	642.9	1511.3	12.0	75.0	445.0	762.8	3225.5	28849.6

A64	L2	T11 (4/8)-T11 (7/8)	67.0	160.1	804.2	1920.7	12.0					
	L3	T11 (8/8)-T11/T12 (3/3)	65.7	165.2	788.7	1982.6	12.0					
	L4	T12 (1/9)-T12 (3/9)	52.7	202.5	474.2	1822.1	9.0					
	L5	T12 (4/9) - T12 (6/9)	31.6	240.6	284.0	2165.0	9.0					
	S	T12 (7/9)	9.6	269.9	231.4	6478.8	21.0					
	Conus tip	L1 (2/9)										
B38	L1	T10/T11 (2/2)-T11 (4/9)	54.1	201.2	812.0	3018.2	15.0	87.0	476.3	719.0	3639.7	30993.1
	L2	T11 (5/9)-T11 (8/9)	61.7	211.3	740.1	2535.1	12.0					
	L3	T11 (9/9)-T12 (1/9)	61.9	218.6	742.3	2623.3	12.0					
	L4	T12 (2/9)-T12 (5/9)	53.4	256.7	640.7	3080.9	12.0					
	L5	T12 (6/9) - T12 (9/9)	33.6	283.0	403.0	3396.1	12.0					
	S	T12/L1 (1/2)	11.2	305.7	301.5	8252.6	24.0					
	Conus tip	L1 (6/9)										
A102	L1	T11 (4/7)-T11/T12 (1/2)	44.2	173.7	662.6	2605.5	15.0	81.0	443.9	690.4	2969.6	28815.8
	L2	T11/T12 (2/2)-T12 (4/8)	53.5	183.0	803.1	2744.9	15.0					
	L3	T12 (5/8)-T12 (8/8)	54.0	204.8	647.8	2457.6	12.0					
	L4	T12/L1 (1/3)-L1 (1/8)	42.1	215.3	505.0	2583.3	12.0					
	L5	L1 (2/8) - L1 (4/2)	22.6	241.3	203.6	2172.1	9.0					
	S	L1 (5/8)	7.0	248.4	147.6	5217.0	18.0					
	Conus tip	L1/L2 (3/3)										
A41	L1	T10/T11 (2/2)-T11 (4/7)	43.2	144.6	745.4	2494.9	17.2	89.7	482.2	734.9	3461.1	21244.4
	L2	T11 (5/7)-T11/T12 (1/2)	56.3	148.6	777.3	2050.6	13.8					
	L3	T11/T12 (2/2)-T12 (3/7)	55.8	182.1	769.7	2513.2	13.8					
	L4	T12 (4/7)-T12 (7/7)	48.0	200.8	663.1	2771.2	13.8					
	L5	T12/L1 (1/2) - L1 (1/8)	26.3	230.9	362.4	3186.1	13.8					
	S	L1 (2/8)	6.9	246.2	143.3	5096.0	17.2					
	Conus tip	L1 (8/8)										
B21	L1	T11 (4/7)-T11 (7/7)	49.1	150.8	677.1	2080.4	13.8	96.6	495.7	747.3	3666.7	23799.5
	L2	T11/T12 (1/3)-T12 (1/7)	57.9	157.6	798.7	2175.5	13.8					
	L3	T12 (2/7)-T12 (5/7)	61.4	199.9	847.7	2758.4	13.8					
	L4	T12 (6/7)-T12/L1 (2/3)	53.5	220.6	738.5	3044.1	13.8					
	L5	T12/L1 (3/3) - L1 (3/8)	30.2	249.8	416.2	3447.1	13.8					
	S	L1 (4/8)	6.1	273.5	188.5	8492.8	27.6					
	Conus tip	L1/L2 (4/4)										

Supplementary Table 2 Statistical Analysis for Global and Local electrode configurations with cathode electrodes located at rostral or caudal portions of the stimulation paddle. Results are divided into comparisons across all muscles and participants (n=12) or individual muscles across participants including left and right soleus (SOL), medial gastrocnemius (MG), tibialis anterior (TA), vastus lateralis (VL), rectus femoris (RF), medial hamstrings (MH), and gluteus maximus (GL), a total of 14 muscles (7 on each side).

Local rostral	Local caudal	Global rostral	Global caudal				
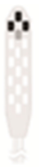	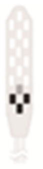	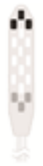	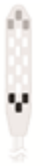	Slope of Recruitment		Activation Threshold	
				Rostral	Caudal	Rostral	Caudal
Across all muscles and individuals	Estimates	Local		1.15±0.39	1.31±0.39	0.64±0.04	0.32±0.04
		Global		2.67±0.39	2.43±0.39	0.3±0.04	0.27±0.04
	Comparisons			Difference	p-value	Difference	p-value
		local: caudal vs rostral		0.16±0.4	t=-0.57, DF=596, p=1	0.32±0.02	t=-13.17, DF=610, p<.0001
		global: caudal vs rostral		-0.23±0.4	t=0.39, DF=596, p=1	0.02±0.02	t=-1.00, DF=610, p=1
		rostral: global vs local		1.52±0.38	t=3.98, DF=596, p=0.0005	0.35±0.03	t=-11.36, DF=610, p<.0001
		caudal: global vs local		1.13±0.38	t=3.98, DF=596, p=0.0182	0.05±0.03	t=-0.59, DF=610, p=0.7513
		global rostral vs local caudal		1.36±0.44	t=3.06, DF=596, p=.0137	0.02±0.04	t=-9.97, DF=610, p=1
		global caudal vs local rostral		1.28±0.44	t=2.9, DF=596, p=0.0235	0.37±0.04	t=-9.97, DF610, p<0.0001
GL	Estimates	Local		0.97±0.72	1.37±0.69	0.64±0.04	0.35±0.04
		Global		2.51±0.69	1.68±0.69	0.34±0.04	0.31±0.04
	Comparisons	local: caudal vs rostral		0.4±0.92	1	0.29±0.04	<.0001
		global: caudal vs rostral		-0.83±0.9	1	0.03±0.04	1
		rostral: global vs local		1.54±0.91	0.5542	-0.3±0.04	<.0001
		caudal: global vs local		0.31±0.89	1	0.04±0.04	1
		global rostral vs local caudal		1.14±0.92	1	0.02±0.05	1
		global caudal vs local rostral		0.7±0.94	1	0.33±0.05	<.0001
MG	Estimates	Local		0.69±0.7	2.37±0.69	0.68±0.04	0.27±0.04
		Global		2.85±0.72	3.6±0.7	0.27±0.04	0.23±0.04
	Comparisons	local: caudal vs rostral		1.68±0.91	0.3938	-0.41±.04	<.0001
		global: caudal vs rostral		0.75±0.93	1	0.03±0.04	1

		rostral: global vs local	2.16±0.92	0.116	0.41±0.04	<.0001
		caudal: global vs local	1.23±0.9	1	0.04±0.04	1
		global rostral vs local caudal	0.48±0.94	0.0121	0±0.05	<.0001
		global caudal vs local rostral	2.91±0.94	0.0121	0.45±0.05	<.0001
MH	Estimates	Local	1.41±0.69	0.93±0.7	0.54±0.04	0.31±0.04
		Global	4.48±0.69	1.82±0.69	0.29±0.04	0.26±0.04
	Comparisons	local: caudal vs rostral	0.48±0.91	1	0.23±0.04	<.0001
		global: caudal vs rostral	-2.66±0.9	0.0198	0.03±0.04	1
		rostral: global vs local	3.06±0.89	0.0038	0.25±0.04	<.0001
		caudal: global vs local	0.88±0.9	0.0198	0.05±0.04	1
		global rostral vs local caudal	3.54±0.93	1	0.03±0.05	<.0001
		global caudal vs local rostral	0.4±0.92	1	0.28±0.05	1
RF	Estimates	Local	0.98±0.69	0.75±0.69	0.58±0.04	0.39±0.04
		Global	2.01±0.69	2.34±0.69	0.32±0.04	0.31±0.04
	Comparisons	local: caudal vs rostral	-0.24±0.9	1	0.19±0.04	<.0001
		global: caudal vs rostral	0.33±0.9	1	0.01±0.04	1
		rostral: global vs local	1.03±0.89	1	0.26±0.04	<.0001
		caudal: global vs local	1.6±0.89	0.4425	0.08±0.04	0.2931
		global rostral vs local caudal	1.27±0.92	1	0.07±0.05	0.7772
		global caudal vs local rostral	1.36±0.92	0.8386	0.27±0.05	<.0001
SOL	Estimates	Local	1.01±0.69	1.5±0.7	0.73±0.04	0.27±0.04
		Global	2.24±0.7	3.25±0.69	0.27±0.04	0.28±0.04
	Comparisons	local: caudal vs rostral	0.49±0.91	1	0.45±0.04	<.0001
		global: caudal vs rostral	1.01±0.91	1	0.01±0.04	1
		rostral: global vs local	1.23±0.9	1	0.46±0.04	<.0001
		caudal: global vs local	1.75±0.9	0.3148	0±0.04	1
		global rostral vs local caudal	0.74±0.94	1	0.01±0.05	1
		global caudal vs local rostral	2.24±0.92	0.0903	0.45±0.05	<.0001
TA	Estimates	Local	1.44±0.72	1.53±0.69	0.75±0.04	0.3±0.04
		Global	1.78±0.7	2.27±0.7	0.3±0.04	0.25±0.04
	Comparisons	local: caudal vs rostral	0.09±0.92	1	0.45±0.04	<.0001
		global: caudal vs rostral	0.49±0.92	1	0.05±0.04	1
		rostral: global vs local	0.34±0.92	1	0.46±0.04	<.0001
		caudal: global vs local	0.74±0.9	1	0.05±0.04	1
		global rostral vs local caudal	0.25±0.93	1	0±0.05	1

		global caudal vs local rostral	0.83±0.95	1	-0.5±0.05	<.0001
VL	Estimates	Local	1.55±0.7	0.72±0.69	0.58±0.04	0.34±0.04
		Global	2.8±0.69	2.09±0.69	0.31±0.04	0.27±0.04
	Comparisons	local: caudal vs rostral	-0.71±0.9	1	0.04±0.04	1
		global: caudal vs rostral	-0.71±0.9	1	0.04±0.04	1
		rostral: global vs local	1.25±0.9	0.9918	0.27±0.04	<.0001
		caudal: global vs local	1.37±0.89	0.7465	0.06±0.04	0.8166
		global rostral vs local caudal	2.08±0.92	0.1452	0.02±0.05	1
		global caudal vs local rostral	0.54±0.93	1	0.31±0.05	<.0001

Supplementary Table 3 Pearson's correlation coefficient analysis and p-values for comparing the spinal cord levels targeted by scES stimulation (midline and unilateral electrode contacts) and the normalized activation threshold and slope of the recruitment curve measurements.

	Activation Threshold				Slope			
	Midline		Unilateral		Midline		Unilateral	
	Correlation coefficient	p-value	Correlation coefficient	p-value	Correlation coefficient	p-value	Correlation coefficient	p-value
GL	-0.26187	<.0001	-0.4984	<.0001	-0.0888	0.0802	-0.10875	0.3132
RF	-0.43953	<.0001	-0.70963	<.0001	0.30689	<.0001	0.15836	0.1383
VL	-0.22959	<.0001	-0.58443	<.0001	0.26063	<.0001	0.34874	0.0008
MH	-0.2736	<.0001	-0.43844	<.0001	-0.08415	0.0945	-0.03839	0.721
TA	0.51604	<.0001	0.61985	<.0001	-0.1118	0.0267	-0.33638	0.0014
MG	0.49987	<.0001	0.59032	<.0001	-0.27958	<.0001	-0.26995	0.0105
SOL	0.38137	<.0001	0.67285	<.0001	-0.22224	<.0001	-0.22349	0.0353

MATLAB Codes

Finding the slope of the recruitment curves

```
clear all
close all
clc
%% load relevant data in MAT format for the recruitment curves
for i=1:length(Event_MeanPeakToPeak)
    for j=1:7
        param=[];
        figure('units','normalized','outerposition',[0 0 1 1]),
        [n,m] = max(Event_MeanPeakToPeak{i}{1}(:,1));
        plot(Event_MeanPeakToPeak{i}{1}(1:m,1),Event_MeanPeakToPeak{i}{1}(1:m,j+1),'-o')
        title([Muscle_names(j),Total_Config_names_array(i)])
        hold on
        [a1,b1]=find(Event_MeanPeakToPeak{i}{1}(1:m,1)<=Voltage(i,j));
        plot(Voltage(i,j),Event_MeanPeakToPeak{i}{1}(a1(end),j+1) ,'*','color','g')
        while isempty(param)
            [X,Y] = ginput(1);
            [a,b]=find(Event_MeanPeakToPeak{i}{1}(1:m,j+1)>=Y);

            try
                param=sigm_fit(Event_MeanPeakToPeak{i}{1}(a1(end):a(1)-
1,1),Event_MeanPeakToPeak{i}{1}(a1(end):a(1)-1,j+1),[],[],1);
            catch
            end
        end
        slope(i,j)=param(4);

    end
    close all
    for j=9:15
        param=[];
        figure('units','normalized','outerposition',[0 0 1 1]),
        [n,m] = max(Event_MeanPeakToPeak{i}{1}(:,1));
        plot(Event_MeanPeakToPeak{i}{1}(1:m,1),Event_MeanPeakToPeak{i}{1}(1:m,j+1),'-o')
        title([Muscle_names(j),Total_Config_names_array(i)])
        hold on
        [a1,b1]=find(Event_MeanPeakToPeak{i}{1}(1:m,1)<=Voltage(i,j));
        plot(Voltage(i,j),Event_MeanPeakToPeak{i}{1}(a1(end),j+1) ,'*','color','g')
        while isempty(param)
            [X,Y] = ginput(1);
            [a,b]=find(Event_MeanPeakToPeak{i}{1}(1:m,j+1)>=Y);

            try
                param=sigm_fit(Event_MeanPeakToPeak{i}{1}(a1(end):a(1)-
1,1),Event_MeanPeakToPeak{i}{1}(a1(end):a(1)-1,j+1),[],[],1);
            catch
            end
        end
        slope(i,j-1)=param(4);
    end
    close all
end
```

Finding activation threshold from motor evoked potentials

```
clear all
close all
clc
%% load relevant data in MAT format for the motor evoked responses

for i=1:length(Event_MeanPeakToPeak)
    for j=1:length(Muscle_names)
        figure('units','normalized','outerposition',[0 0 1 1]),
        [n,m] = max(Event_MeanPeakToPeak{i}{1}(:,1));
        plot(Event_MeanPeakToPeak{i}{1}(1:m,1),Event_MeanPeakToPeak{i}{1}(1:m,j+1),'-o')
        title([Muscle_names(j),Total_Config_names_array(i)])
        [X,Y] = ginput(1);
        Voltage(i,j)=round(X,1);
        Peak(i,j)=Y;
        figure('units','normalized','outerposition',[-1 0 1 1])


        a1 = find(Event_MeanPeakToPeak{i}{1}(1:m,1)>=Voltage(i,j));
        a=a1(1);


        if a-10>=1
            inx = [-10,-9,-8,-7,-6,-5,-4,-3,-2,-1,0,1,2,3,4,5,6,7,8,9];
            for k=1:20
                subplot(5,4,k)
                if Total_events_sample{i}{1}(a+inx(k))>0
                for kk = Total_events_sample{i}{1}(a+inx(k)):Total_events_sample{i}{1}(a+inx(k) +1)-1
                    plot(Total_Heatmap{i}{1}(kk,1:round(size(Total_Heatmap{i}{1},2)/8),j),'color',[0 0
0],'LineWidth',1);
                    hold on
                    xlim([0 length(Total_Heatmap{i}{1}(kk,1:round(size(Total_Heatmap{i}{1},2)/8),j))])
                end
                else
                    for kk = 1:Total_events_sample{i}{1}(a+inx(k) +1)-1
                    plot(Total_Heatmap{i}{1}(kk,1:round(size(Total_Heatmap{i}{1},2)/8),j),'color',[0 0
0],'LineWidth',1);
                    hold on
                    xlim([0 length(Total_Heatmap{i}{1}(kk,1:round(size(Total_Heatmap{i}{1},2)/8),j))])
                    end
                end
                if inx(k)==0
                title(Event_MeanPeakToPeak{i}{1}(a+inx(k),1),'color',[1 0 0])
                else
                    title(Event_MeanPeakToPeak{i}{1}(a+inx(k),1))
                end
            end
        else
            for k=1:20
                subplot(5,4,k)
                if Total_events_sample{i}{1}(k)>0
                for kk = Total_events_sample{i}{1}(k):Total_events_sample{i}{1}(k+1)-1
                    plot(Total_Heatmap{i}{1}(kk,1:round(size(Total_Heatmap{i}{1},2)/8),j),'color',[0 0
0],'LineWidth',2);
                    hold on
                    xlim([0 length(Total_Heatmap{i}{1}(kk,1:round(size(Total_Heatmap{i}{1},2)/8),j))])
                end
                else
                    for kk = 1:Total_events_sample{i}{1}(k+1)-1
                    plot(Total_Heatmap{i}{1}(kk,1:round(size(Total_Heatmap{i}{1},2)/8),j),'color',[0 0
0],'LineWidth',2);
                    hold on
                    xlim([0 length(Total_Heatmap{i}{1}(kk,1:round(size(Total_Heatmap{i}{1},2)/8),j))])
                    end
                end
                if k==a
                    title(Event_MeanPeakToPeak{i}{1}(k,1),'color',[1 0 0])
                else
                title(Event_MeanPeakToPeak{i}{1}(k,1))
                end
            end
        end
        figure('units','normalized','outerposition',[0 0 1 1]),
        plot(Event_MeanPeakToPeak{i}{1}(1:m,1),Event_MeanPeakToPeak{i}{1}(1:m,j+1),'-o')
        hold on
```

```
        plot(Voltage(i,j),Event_MeanPeakToPeak{i}{1}(a,j+1),'o','color',[1 0 0])
        title([Muscle_names(j),Total_Config_names_array(i)])
        [X,Y] = ginput(1);
        Voltage(i,j)=round(X,1);
        Peak(i,j)=Y;

        close all
    end
end
```

Calculating the percentage of volumetric coverage of lumbosacral enlargement by the scES paddle array

```
clear all
close all
clc
% load manual segmentation of MRI scans of spinal cord in nifti files
% identify the axial slice number for top of sc-L1 from manual nerve root tracing and place that number
in TopOfL1Slice variable
% TopOfL1Slice
% x1 = find the axial slice number for the top of the paddle electrode based on integration of MRI and
X-ray images
% x2 = find the axial slice number for the bottom of the paddle electrode based on integration of MRI
and X-ray images


Cord = flip(ROI.img,3);
a = unique(Cord);
Spine_vol = double(Cord==a(3));

for i=1:size(Spine_vol,3)
    manual_vol(i,1) = nnz(Spine_vol(:,:,i))*ROI.hdr.dime.pixdim(2)*ROI.hdr.dime.pixdim(3);
end

LSE = sum(manual_vol(TopOfL1Slice:end,1))*ROI.hdr.dime.pixdim(4); %% volume of the cord in mm^3 for the
Lumbosacral enlargement part

x2 = min(x2,size(manual_vol,1));
LSE_electrode = sum(manual_vol(x1:x2,2))*ROI.hdr.dime.pixdim(4); %% volume of the cord in mm^3 for the
Lumbosacral enlargement part

Percent_Volume_Coverage = (LSE_electrode(2,1)/LSE(2,1))*100
```

3D visualization

```
clear all
close all
clc
% load manual segmentation of MRI scans of spinal cord in nifti files in variable V
% find the axial slice numbers for the beginning of spinal cord levels L1-S from manual nerve root
tracing of MRI scans and add them to Cord_segments = [1,7,12,17,21,25,29,33,38];

cord = double(V.img(:,:,:));
cord = flip(cord,3);
a = unique(cord);

mask = zeros(size(cord));
mask(cord==a(2)) = 1; % find cord segmentation
mask(cord==a(3)) = 2; % find CSF segmentation
mask(cord==a(5)) = 3; % find nerve roots segmentation

V1=double(mask==1);
V1=smooth3(V1);

V2=double(mask==2);
V2=smooth3(V2);

V3=double(mask==3);
```

```
V3=smooth3(V3);

[Face1 Vers1]=isosurface(V1);
[Face2 Vers2]=isosurface(V2,.2);
[Face3 Vers3]=isosurface(V3,0.3);

h=figure('Position',[0    32   389   954]);
patch('Faces',Face1,'Vertices',Vers1,'FaceColor',[.8 .8 .8],'Edgecolor','none');
alpha(.25);
view(-90,7)
hold
light
lightangle(-45,30)
lighting phong;
set(gca, 'visible', 'off')
set(gcf,'Color',[1 1 1])
patch('Faces',Face3,'Vertices',Vers3,'FaceColor',[0.95 0.87 0.53],'Edgecolor','none');

alpha(.3);

for i=1:2:length(Cord_segments)-1
V22=zeros(size(V2));
V22(:,:,end - (Cord_segments(i+1)-1):end - (Cord_segments(i)-1))=V2(:,:,end - (Cord_segments(i+1)-
1):end - (Cord_segments(i)-1));
[Face22 Vers22]=isosurface(V22,.1);
patch('Faces',Face22,'Vertices',Vers22,'FaceColor',[0.93 0.84 0.84],'Edgecolor','none');
clear V22 Face22 Vers22
end
for i=2:2:length(Cord_segments)-1
V22=zeros(size(V2));
V22(:,:,end - (Cord_segments(i+1)-1):end - (Cord_segments(i)-1))=V2(:,:,end - (Cord_segments(i+1)-
1):end - (Cord_segments(i)-1));
[Face22 Vers22]=isosurface(V22,.1);
patch('Faces',Face22,'Vertices',Vers22,'FaceColor',[0.93 0.84 0.84],'Edgecolor','none');
clear V22 Face22 Vers22
end

hold
light
lighting phong;

paddle_placement = [19,34];

V22=zeros(size(V2));
V22(:,:,end - (paddle_placement(2)-1):end - (paddle_placement(1)-1))=V2(:,:,end - (paddle_placement(2)-
1):end - (paddle_placement(1)-1));
[Face22 Vers22]=isosurface(V22,.1);
patch('Faces',Face22,'Vertices',Vers22,'FaceColor',[0.93 0.90 0.84],'Edgecolor','none');

filename = 'testAnimated.gif';
axis tight manual
```
